# Supplementary material for: Changes in perineuronal net and parvalbumin expression in the orbitofrontal cortex of male Wistar rats following repeated fentanyl administration
Source: Front Neurosci. 2026 Jul 8;20:1821692. doi: 10.3389/fnins.2026.1821692 (PMC13390114; doi:10.3389/fnins.2026.1821692)
Supplement: Supplementary file 1 [file Data_Sheet_1.pdf]

Supplementary Materials for:

Changes in perineuronal net and parvalbumin expression in the orbitofrontal cortex of male Wistar rats following repeated fentanyl administration

Mariana I. H. Dejeux[1], Sarah S. Jewanee[1], Samuel Moutos[1], Arjun Trehan[1], Melody Golbarani[1], Joanne Kwak[1], Evan Farach[1], Nathan Cheng[1], Sri V. Kasaram[1], Aliyah Ogden[1], Sophia Wright[1], Benjamin A. Schwartz[1], Jacques D. Nguyen*[1]

[1]Department of Psychology and Neuroscience; Baylor University; Waco, TX USA

*Corresponding Author

Supplementary Figure 1

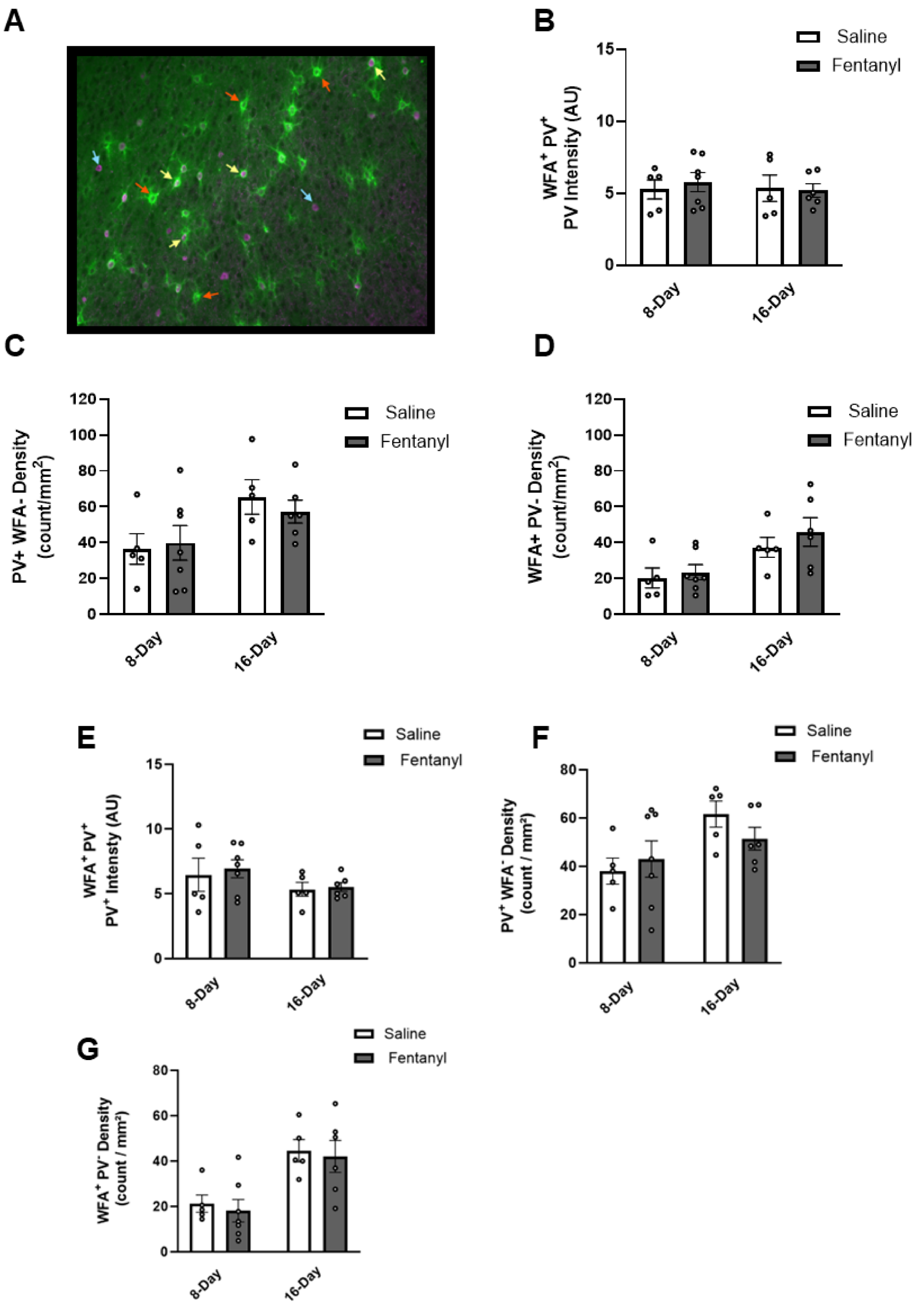

Supplementary Figure 1. $WFA^{+}PV^{+}$ cells in the Orbitofrontal Cortex. (A) Representative images of combined $WFA^{+}PV^{+}$ stained tissue in the Orbitofrontal Cortex (B) PV^{+} Intensity (AU) for $WFA^{+}PV^{+}$ cells in the VO of 8-day and 16-day groups. (C) $PV^{+}WFA^{-}$ Density in the VO of 8-day and 16-day groups. (D) $WFA^{+}PV^{-}$ Density in the VO of 8-Day and 16-Day groups (E) PV^{+} Intensity (AU) for $WFA^{+}PV^{+}$ cells in the LO of 8-day and 16-day groups. (C) $PV^{+}WFA^{-}$ Density in the LO of 8-day and 16-day groups. (D) $WFA^{+}PV^{-}$ Density in the VO of 8-Day and 16-Day groups.

Supplementary Methodology: WFA and PV Quantification

For the purpose of quantification, tissue was imaged using a fluorescence microscope (Olympus IX-81) with a DP81, Peltier cooled 12.5MP digital camera and using a 4X objective. WFA^+ images were imaged using a GFP-filter (ext. 450-490nm, em. 500-550nm) at 166.7ms exposure. PV^+ cells were imaged using a CY5-filter (ext. 605-645nm, em. 650-710nm) at a 250ms exposure.

Images were uploaded onto polygon AI. Both detection sensitivity and overlap removal were set to 80. Image split was set to 3. PNNs and PV^+ cells were detected according to the "Biomarker-Specific Detection Models" provided on the Polygon AI software. False negatives and positives were corrected as outline below. A single experimenter was chosen to quantify all samples represented in this study to maintain consistency in the parameters that were used to identify both PNNs and PV+ cells.

<u>WFA:</u> To account for potential differences in PNN morphology (Banovac et al., 2024; Dauth et al., 2016; Lensjø et al., 2017) the experimenter used the following guidelines [1] difference in color shade (brighter green) between the PNN, and background staining was present due to a difference in staining intensity. [2] At least 50% of the PNN was surrounding a soma, identified as a darkened, circular center.

<u>PV:</u> PV^+ interneurons were quantified based on the [1] difference in color shade (brighter purple) between the PV, and background staining was present due to a difference in staining intensity. [2] circular shape

<u>WFA^+ PV^+ cells</u> were identified using the following steps:

The experimenter uploaded WFA^+ PV^+ colocalized images onto polygon AI The "import ROI" feature on polygon AI was used to upload WFA^+ PNNs that were identified from WFA^+ images. The experimenter deleted any ROIs that did not fit the following criteria [1] The identified soma must be stained purple. [2] At least 50% of the soma must be surrounded by the WFA^+ PNN

To maintain consistency across samples, WFA+ intensity was only obtained from images that were captured at 166.7ms and PV intensity was only obtained from images that were captured at 250 ms. Intensities were not recorded from colocalized images, instead the identified ROIs for WFA^+ PV^+ cells were imported back onto the WFA^+ and PV^+ images separately and the intensity values from these images were used data showing WFA^+ intensity for WFA^+ PV^+ cells and PV^+ intensity for WFA^+ PV^+ cells.

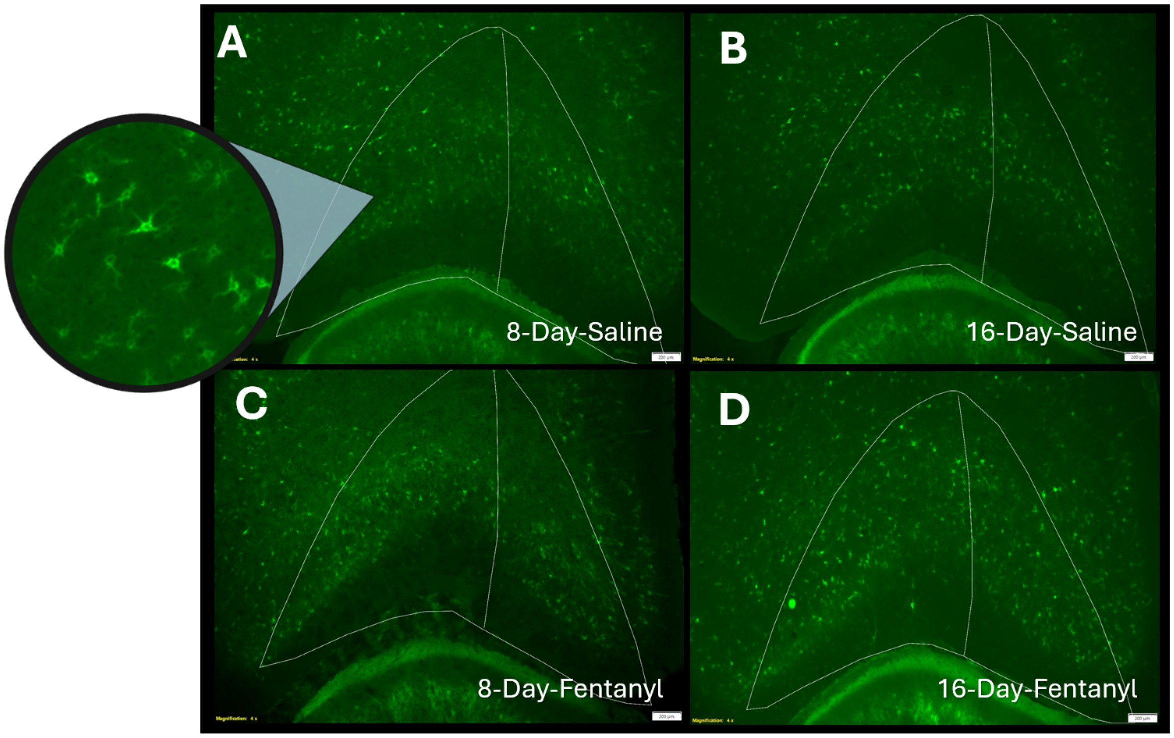

Supplementary Figure 2. WFA^{+} PNNs in the OFC. (A) Representative image of WFA^{+} PNNs in the OFC of 8-Day saline group (B) Representative image of WFA+ PNNs in the OFC of 16-Day saline group (C) Representative image of WFA^{+} PNNs in the OFC of 8-Day fentanyl group (D) Representative image of WFA^{+} PNNs in the OFC of 16-Day fentanyl group. Scale bar: 200 μm

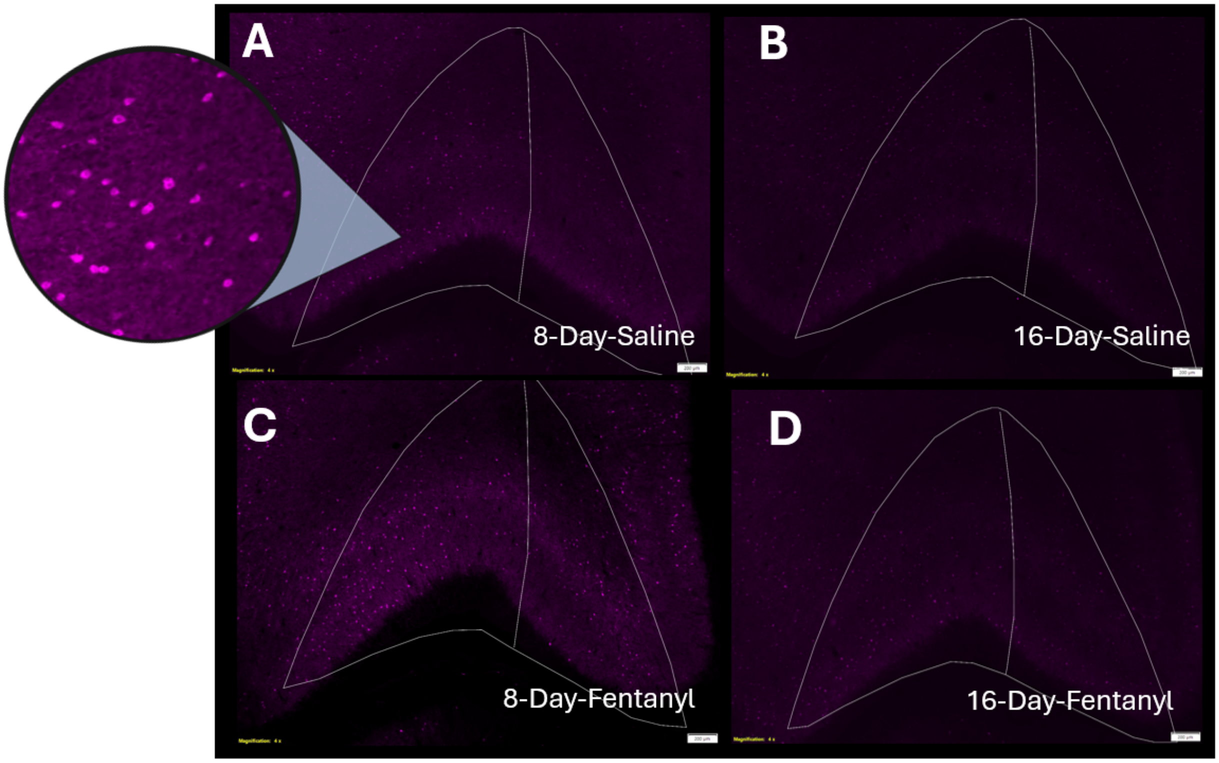

Supplementary Figure 3. PV$^+$ interneurons in the OFC. (A) Representative image of PV$^+$ cells in the OFC of 8-Day saline group (B) Representative image of PV$^+$ cells in the OFC of 16-Day saline group (C) Representative image of PV$^+$ cells in the OFC of 8-Day fentanyl group (D) Representative image of PV$^+$ cells PNNs in the OFC of 16-Day fentanyl group. Scale bar: 200 μm

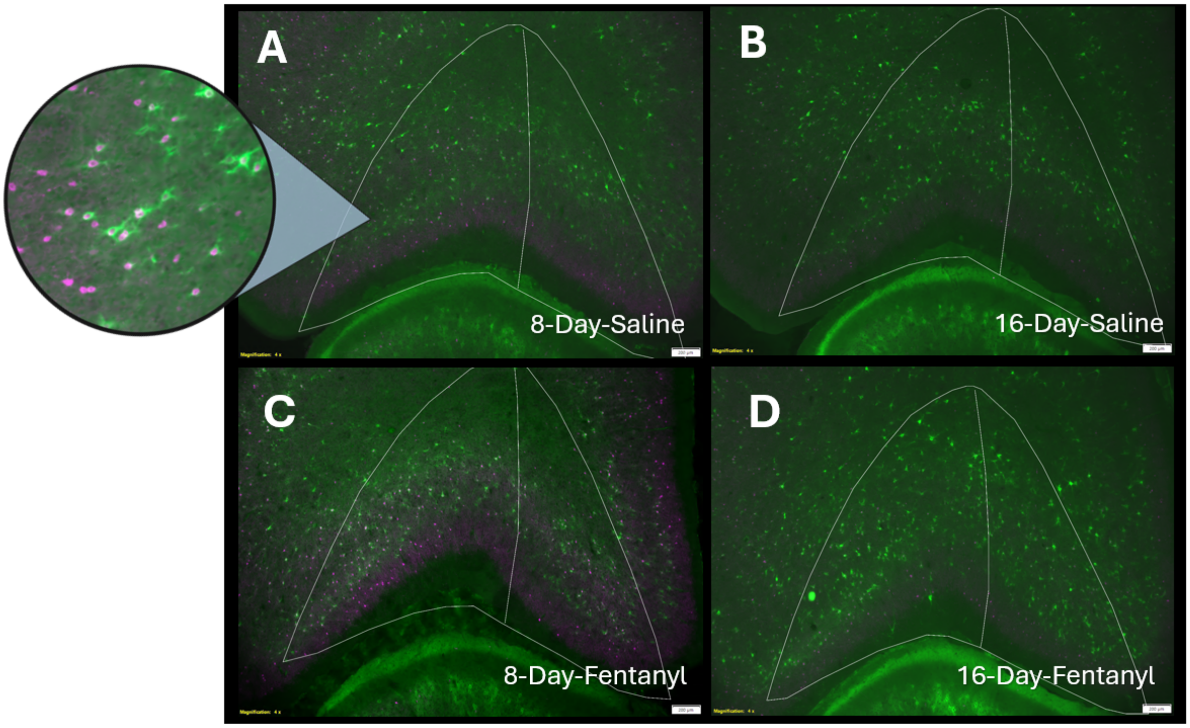

Supplementary Figure 4. WFA^+ and PV^+ cells in the OFC. (A) Representative image of WFA^+ and PV^+ cells in the OFC of 8-Day saline group (B) Representative image of WFA^+ and PV^+ cells in the OFC of 16-Day saline group (C) Representative image of WFA^+ and PV^+ cells in the OFC of 8-Day fentanyl group (D) Representative image of WFA^+ and PV^+ cells in the OFC of 16-Day fentanyl group. Scale bar: 200 μm

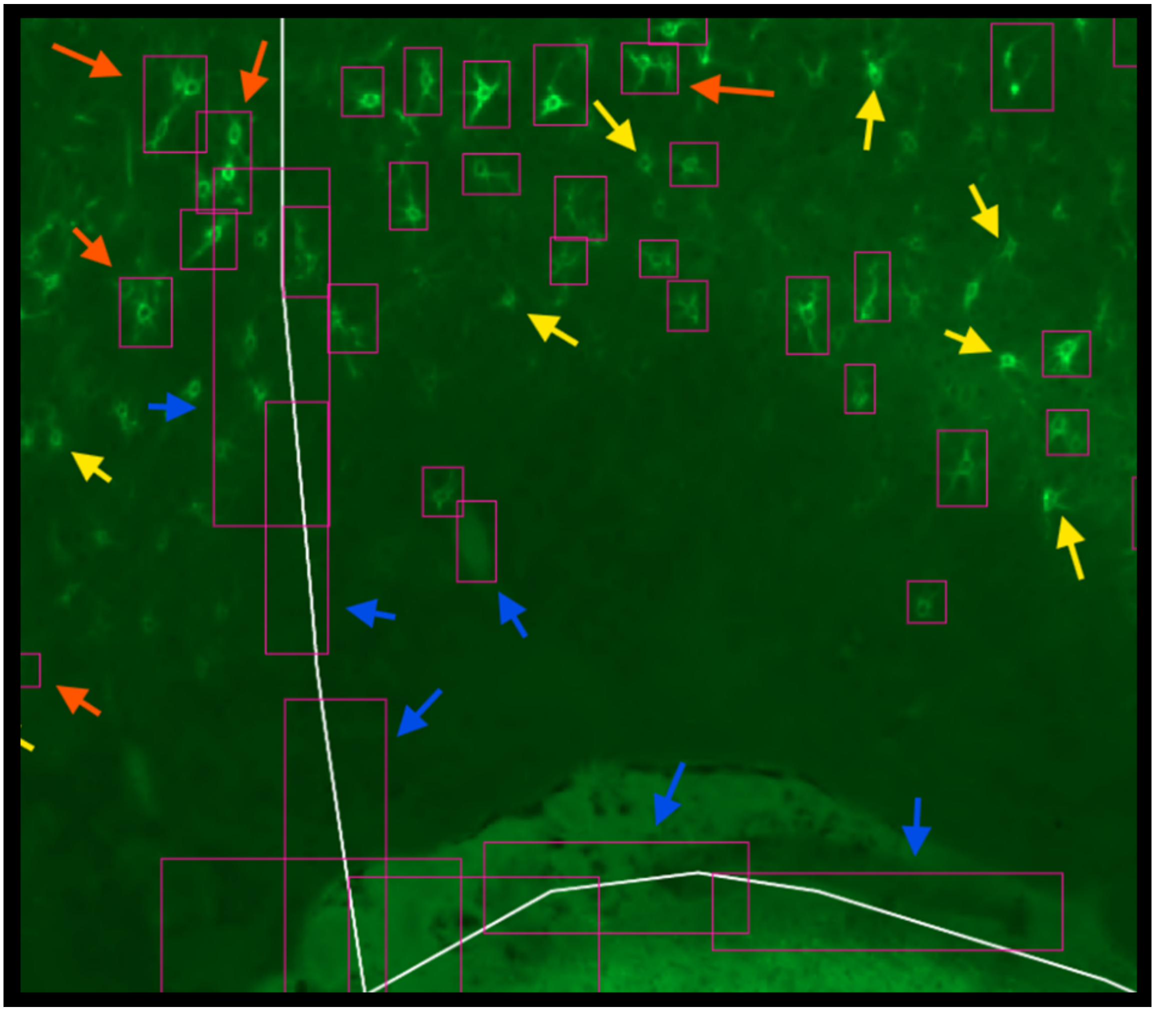

Supplementary Figure 5. Representative image illustrating the quantification of WFA+ images. Blue arrows show identified false positives. These were ROIs that were placed around image artifacts that did fit the identified criteria. Additionally, any ROIs that were outside of the outline OFC were deleted. Red arrows show ROIs that were placed on PNNs but needed to be corrected. These corrections occurred when the ROI was placed around multiple PNNs or the ROI was not fully surrounding the PNN.Yellow arrows show some examples of the identified false negatives.

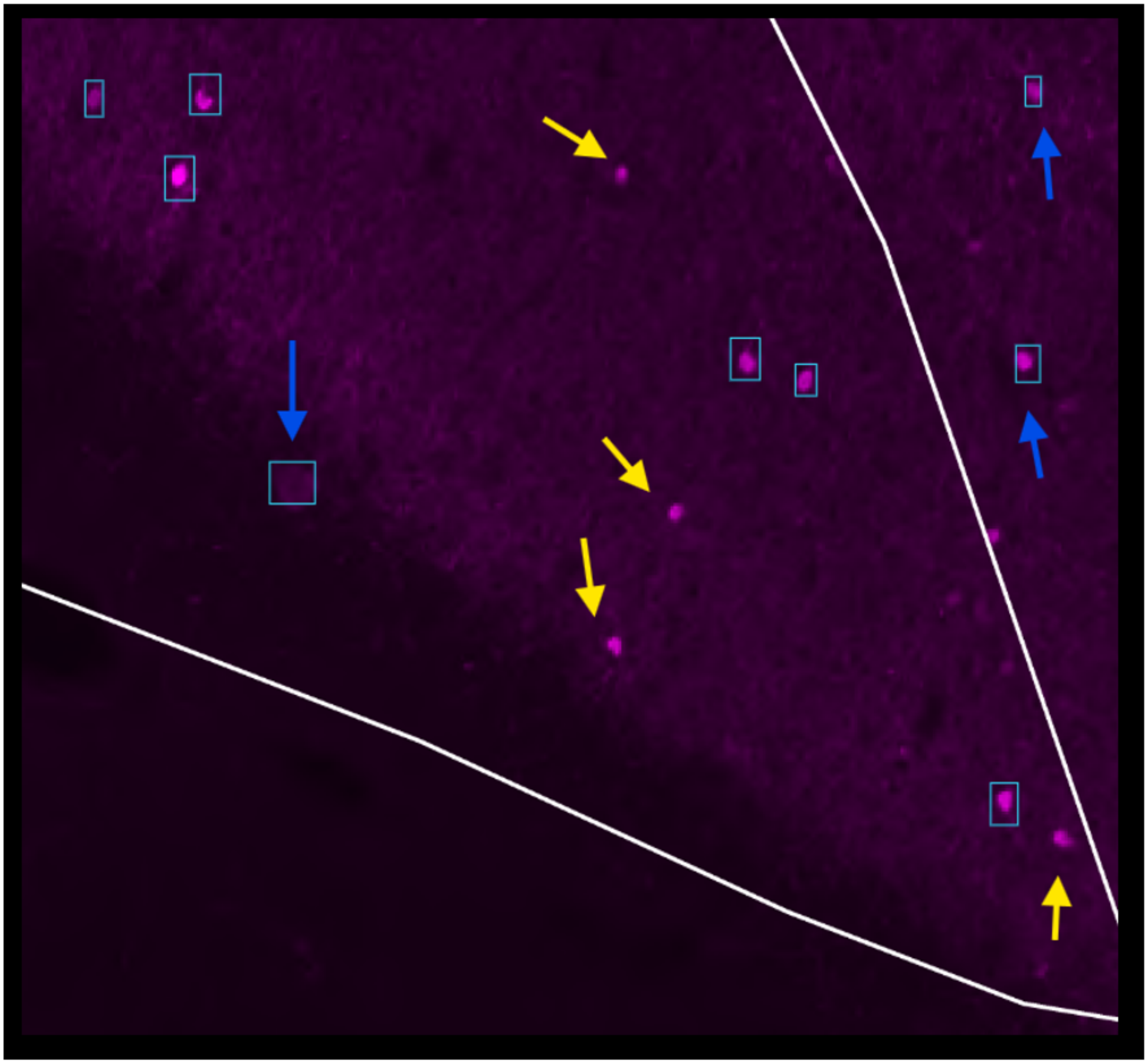

Supplementary Figure 6. Representative image illustrating the quantification of PV+ images. Blue arrows show identified false positives. These were ROIs that were placed around image artifacts that did not fit the identified criteria. Additionally, any ROIs that were outside of the outline OFC were deleted. Yellow arrows show some examples of the identified false negatives. PNNs were identified according to their round shape.

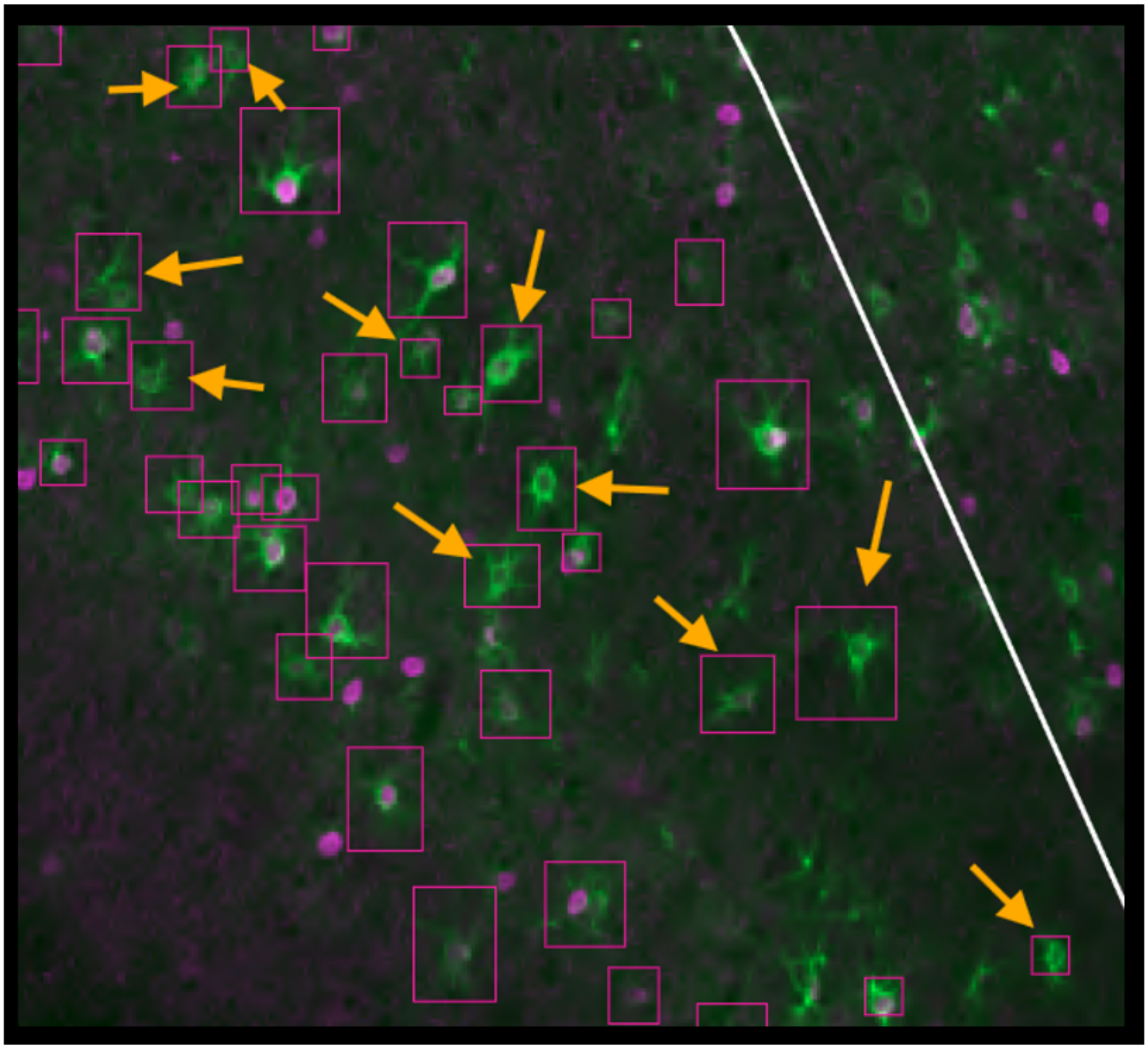

Supplementary Figure 7. Representative image illustrating the quantification of WFA+ PV+ images. Orange arrows show WFA+ PNNs that are not PV+; these ROIs were removed.

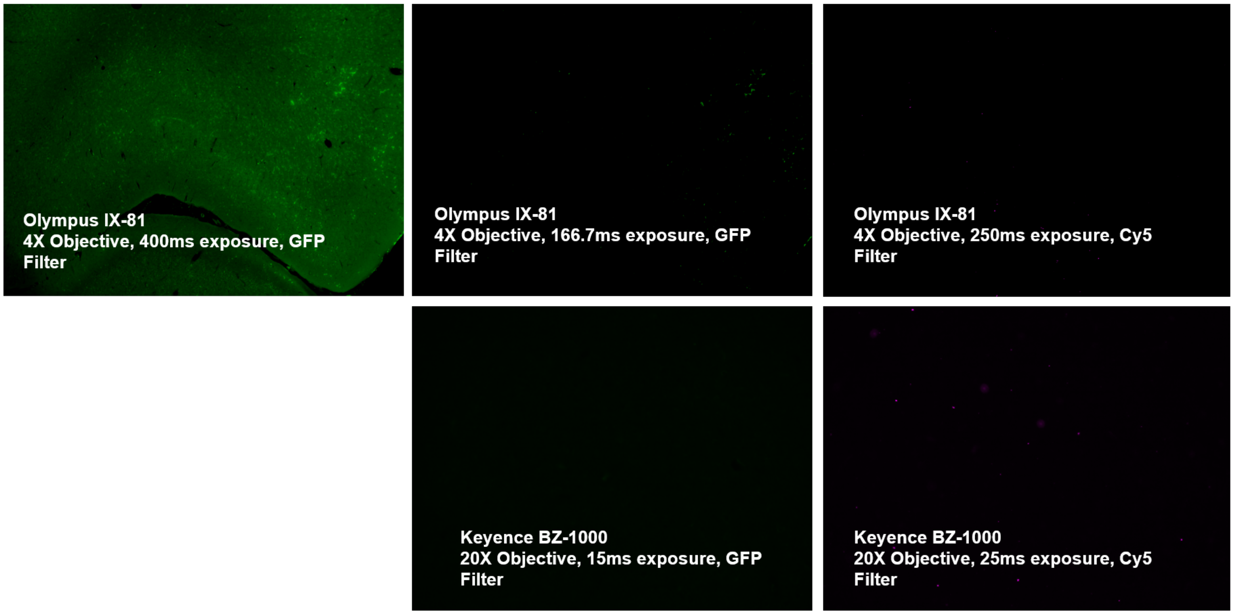

Supplementary Figure 8. We imaged tissue that underwent several PBS washes and a 2 hour incubation with the same secondary that was used for our experiments (Goat anti-Rabbit IgG (H+L) Cross-Adsorbed Secondary Antibody, Alexa Fluor 647). Imaging was done with both the fluorescence microscope (Olympus IX-81), using the same settings as our quantified tissue. We included an image captured at 400ms to show that we imaged the correct region. Imaging was also done with the fluorescence microscope (Keyence BZ-1000) used for representative imaging, using the same settings that were set up for our representative images.
